# *DcSto*: carrot *Stowaway*-like elements are abundant, diverse, and polymorphic

**DOI:** 10.1007/s10709-013-9725-6

**Published:** 2013-06-18

**Authors:** Alicja Macko-Podgorni, Anna Nowicka, Ewa Grzebelus, Philipp W. Simon, Dariusz Grzebelus

**Affiliations:** 1 Department of Genetics, Plant Breeding and Seed Science, University of Agriculture in Krakow, Al. 29 Listopada 54, 31-425 Kraków, Poland; 2 USDA-ARS Vegetable Crops Research Unit, Department of Horticulture, University of Wisconsin-Madison, 1575 Linden Drive, Madison, WI 53706 USA

**Keywords:** Transposable elements, MITE, *Stowaway*, Carrot, Polymorphism

## Abstract

We investigated nine families of *Stowaway*-like miniature inverted-repeat transposable elements (MITEs) in the carrot genome, named *DcSto*1 to *DcSto*9. All of them were AT-rich and shared a highly conserved 6 bp-long TIR typical for *Stowaway*s. The copy number of *DcSto*1 elements was estimated as ca. 5,000 per diploid genome. We observed preference for clustered insertions of *DcSto* and other MITEs. Distribution of *DcSto*1 hybridization signals revealed presence of *DcSto*1 clusters within euchromatic regions along all chromosomes. An arrangement of eight regions encompassing *DcSto* insertion sites, studied in detail, was highly variable among plants representing different populations of *Daucus carota*. All of these insertions were polymorphic which most likely suggests a very recent mobilization of those elements. Insertions of *DcSto* near carrot genes and presence of putative promoters, regulatory motifs, and polyA signals within their sequences might suggest a possible involvement of *DcSto* in the regulation of gene expression.

**Electronic supplementary material:**

The online version of this article (doi:10.1007/s10709-013-9725-6) contains supplementary material, which is available to authorized users.

## Introduction

Transposable elements (TEs), DNA segments capable of changing their chromosomal position, are present in genomes of almost all living organisms. In plants, TEs can constitute from ca. 10 % of *Arabidopsis thaliana* genome (Arabidopsis Genome Initiative 2000) to 85 % of the B73 maize genome (Schnable et al. 2009). Based on the mechanism of transposition, TEs are divided into two groups—class I: retrotransposons, and class II: DNA transposons. Retrotransposons transpose via an RNA intermediate (a ‘copy-and-paste’ mechanism) and each transposition event leads to increase of their copy number. DNA transposons (class II) comprise two subclasses, divided with respect to the number of strands that are cleaved during transposition. Class II TEs belonging to subclass 1 are mobilized via a ‘cut-and-paste’ mechanism, where both DNA strands are cleaved at each end during transposition, while mobilization of subclass 2 elements does not require double-strand cleavage (Finnegan 1989; Wicker et al. 2007). Subclass 1 is further divided into two orders, i.e. TIR, characterized by the presence of terminal inverted repeats (TIRs), and *Crypton*, identified in fungi, devoid of TIR sequences.

Miniature inverted-repeat transposable elements (MITEs), are usually the most abundant group of DNA transposons. They are characterized by a small size (>600 bp) and similarity of their TIRs to the termini of related Class II TIR transposons (Wicker et al. 2007). *Stowaway* MITEs were described in maize (Bureau and Wessler 1994) as short (<500 base pairs), AT-rich, having a potential to form secondary structures and forming 2-nt ‘TA’ target site duplications (TSD) upon insertion. Some *Stowaway*s may provide polyadenylation sites and *cis*-acting regulatory regions to adjacent genes. *Stowaway*s were identified in both monocots and dicots (Feschotte et al. 2003). Due to similarity of TIRs (5′-CTG CCT CCR T-3′, where R stands for A or G) and TSD, it was suggested that *Stowaway* elements could be mobilized *in trans* by transposases encoded by *Tc1/mariner*-like DNA transposons (Feschotte and Wessler 2002; Feschotte et al. 2003, 2005; Macas et al. 2005). Also, it was shown that a single source of transposase can interact with *Stowaway*s belonging to several distinct families. The transposase has a relatively weak binding specificity and the function of TIRs might by suppressed or enhanced by an internal sequence of the element (Feschotte et al. 2005; Yang et al. 2009). Excision of *mariner*s and *Stowaway*s leaves a characteristic footprint, which is element-specific, corresponding to the 5′ and 3′ TIR nucleotides and can be of variable length (Lampe et al. 1996). Thus, a previously occupied locus can be distinguished from the ancestral empty site. *Stowaway* elements are relatively hypomethylated and frequently occur in genic regions (Mao et al. 2000; Takata et al. 2007). Recently, an active *Stowaway* element was identified in potato. Mobilization took place in the course of leaf protoplast cultures and caused somaclonal variation of skin color of potato tubers (Momose et al. 2010). Availability of genomic sequences significantly increased the number of TE families identified and facilitated their classification. Nevertheless, in species for which sequence data are restricted, new transposons are often identified following the analysis of knock-out mutations caused by TE insertions into coding regions.

Carrot is one of the most important vegetable crops and a major source of carotenoids that are precursors of vitamin A. However, sequencing data for carrot are not extensive and little is known about the organization of the repetitive portion of the carrot genome, including transposable elements. To date, only two families of TEs, i.e. *Tdc1* of CACTA superfamily (Ozeki et al. 1997; Itoh et al. 2003) and *DcMaster* of PIF/*Harbinger* superfamily (Grzebelus et al. 2007), the latter associated with a family of *Tourist*-like MITEs named *Krak* (Grzebelus and Simon 2009), were identified in the carrot genome. In the present study, we investigated carrot *Stowaway*-like MITEs, *DcSto* (*Daucus carota* *Stowaway*-like), estimated their copy number, identified local rearrangements created upon insertion that pointed at putative functional implications of their presence on adjacent coding regions.

## Materials and methods

### Plant materials

DNA was extracted from plants representing carrot diversity, comprising cultivated carrots of different origin and breeding history (supplementary Table 1) previously used for carrot genetic diversity study (Baranski et al. 2011). Full-length *DcSto*1 elements were amplified from the genomic DNA of three unrelated cultivated carrots (8503B, 493B, 70349-F2), three wild carrots (*Daucus carota* subsp. *carota*, *D. carota* subsp. *commutatus*, *D. carota* subsp. *gummifer*) and other closely related *Daucus* species (*D. capillifolius*, *D. sahariensis*).

### DNA isolation, cloning and sequencing

Total genomic DNA was isolated from fresh, young leaves using commercial DNeasy Plant Mini Kit (Qiagen) following a manufacturer’s protocol. PCR products separated on agarose gels were cut out, purified with Wizard SV Gel and PCR Clean-Up (Promega), cloned into pGEM-T (Promega) and transformed into *Escherichia coli*, strain DH10B or PEAK™ Efficiency Competent Cells (GeneMate). From the transformed bacterial cells, identified by blue-white selection, plasmids were extracted using Wizard SV Miniprep kit (Promega). Sequencing reactions were set up with universal primers Sp6 and T7 or PCR-specific primers using Big Dye terminator chemistry (Applied Biosystems) or CEQ™ DTCS Quick Start Kit (Beckman Coulter) as recommended by manufacturer. Sequencing was carried out on ABI 3700 capillary sequencer (Applied Biosystems) or CEQ8000 capillary DNA sequencer (Beckman Coulter).

### Amplification of *DcSto*1 from the Genomic DNA of Carrot

A primer complementary to the TIR sequence of a *DcSto*1 (DcS-TIR) was designed manually based on sequence of *DcSto*1 element identified in the *Rs* locus. To design the DcS-TIR primer, both *Stowaway* characteristic features, 16 bp consensus TIR sequence and the characteristic TSD, were considered. To amplify full-length *DcSto*1 elements the reaction was prepared in 10 μl and contained 20 ng genomic DNA, 1 mM DcS-TIR primer (5′ TAC TCC CTC CGT CCC ACC 3′), 0.25 mM dNTPs (Fermentas), 0.5 U Taq DNA polymerase (Fermentas) and 1× Taq buffer. The amplification profile was as follows: 94 °C (1 min), 30 cycles of 94 °C (30 s), 50 °C (40 s), 68 °C (1 min) and 68 °C (4 min). PCR products were separated in 1 % agarose gels, purified, cloned, and sequenced.

To identify *DcSto* insertion in the *chxb1* gene, intron primers were designed based on the available mRNA sequence (GenBank: DQ192193). A PCR reaction was set up in 10 μl containing 10 ng genomic DNA, 1 mM CHXB1-5′F: 5′CCG AAA TGA TAG CTC GGG TA3′ primer, 1 mM CHXB1-5′R: 5′AGC CTT TGT GGA AGA AAC CA3′primer, 0.25 mM dNTPs (Fermentas), 1 U Taq DNA polymerase (Fermentas) and 1× Taq buffer. The amplification profile was as follows: 94 °C (1 min), 30 cycles of 94 °C (30 s), 56 °C (45 s), 68 °C (3 min) and a final extension step of 68 °C (8 min). Amplified products were separated in 1 % agarose gels, purified, cloned, and sequenced.

### Estimation of the Copy Number of *DcSto* 1

Copy number of *DcSto*1 elements was estimated essentially as proposed by Grzebelus et al. (2006). Two rounds of amplification with DcS-TIR primer were carried out. First PCR was set up to check if at least one element is present per 384-well BAC plate and to confirm the specificity of amplification. For this purpose, pools by plate of B8503 carrot genomic BACs (Cavagnaro et al. 2009) were used. PCR reaction contained 10–30 ng BAC DNA, 1 mM DcS-TIR primer (5′ TAC TCC CTC CGT CCC ACC 3′), 0.25 mM dNTPs (Fermentas), 0.5 U Dream Taq DNA polymerase (Fermentas) and 1× Dream Taq buffer. The thermal profile was as follows: 94 °C (2 min), 30 cycles of 94 °C (30 s), 50 °C (40 s), 68 °C (1 min) and 68 °C (5 min). PCR products were separated in 1 % agarose gels, three products of expected size and all amplicons larger than expected were cut out, purified, cloned, and sequenced. Subsequently, PCR under the same conditions as above was carried out using DNAs of 141 randomly chosen BAC clones. Amplicons were separated in 1 % agarose gels and scored. We estimated the copy number of *DcSto*1 elements in the carrot genome taking into account that the average BAC clone size was 0.121 Mbp (Cavagnaro et al. 2009) and that the 2n carrot genome was approximately 980 Mbp (Bennett and Leith 1995).

### Inverse PCR, Design, and Validation of Site-Specific Primers

Inverse PCR was set up as described by Collins and Weissman (1984). Ca. 100 ng of genomic DNA was digested with 1 U *Taq*I (5′T^CGA3′), *Msp*I (5′C^CGG3′), *EcoR*I (5′G^AATTC3′), or *Nde*I (5′CA^TATG3′) (Fermentas) at 37 °C for 3 h in the total volume of 10 μl and incubated for 20 min at 65 °C for thermal inactivation of the enzyme. Within *DcSto* sequence, restriction sites of the applied enzymes were not present. To achieve intramolecular circularization, 5 μl of each digestion mixture was incubated overnight at 4 °C with 5 U of T4 DNA ligase (Fermantas).

Inverse PCR products were amplified with primers anchored in opposite directions at both ends of the *DcSto*1 element (DcSiPCR-F 5′ CCA CTT CAC CCA CTT TTC CT 3′ and DcSiPCR-R 5′TTT TAG GAA AGT TTT GTA ATG TAA AGA 3′). Primers were designed with Primer3 (v. 0.4.0) (Rozen and Skaletsky 2000) based on the sequence of identified *DcSto* elements. Each 20 μl reaction mixture contained 10 ng self-ligated DNA, 1 mM each primer, 0.25 mM dNTPs (Fermentas), 1 U Taq DNA polymerase (Fermentas) and 1× Taq buffer. The reactions were carried out as follows: 94 °C (1 min), 30 cycles of 94 °C (30 s), 50 °C (45 s), 68 °C (4 min), and a final extension step of 68 °C (10 min). To obtain flanking sequences long enough to design site-specific primers, only products longer than 400 bp were cloned and sequenced.

Local BLAST search was used to mine BAC-end sequences (BES) database with *DcSto*1 sequence as a query (e-value cutoff was 0.01). Identified BES sequences with insertions of *DcSto* elements carrying characteristic TSD, TIR sequence, and for which enough flanking sequence was available, were used for further analysis. Boundary sequences of insertion sites obtained following iPCR or in silico analysis of BES, were used to design site-specific primers with Primer3 (v. 0.4.0) using default parameters.

Site-specific PCR was carried out in 10 μl containing around 20 ng genomic DNA, 1 mM forward and reverse primer, 0.25 mM dNTPs (Fermentas), 0.5 U Taq DNA polymerase (Fermentas), and 1× Taq buffer supplied with MgCl_2_ (Fermentas). Amplification profile was as follows: 94 °C (1 min), 30 cycles of 94 °C (30 s), variable annealing temperature from 55 to 58 °C (depending on the primer combination) (30 s), 68 °C (variable time depending on the primer combination, from 1 to 3 min), and 68 °C (6 min). All primer sequences, the corresponding annealing temperatures and times of elongation are provided in supplementary Table 2. Products were separated on 1 % agarose gels and selected amplicons were sequenced.

### Sequence evaluation and analysis

*DcSto* sequences were aligned using ClustalX (Thompson et al. 1997) and manually edited in BioEdit (Hall 1999). Genetic distances were calculated with Dnadist in PHYLIP (Felsenstein 1996) using Kimura two-parameter model of nucleotide substitution, Neighbor Joining (NJ) tree was produced with Neighbor and plotted with TreeView (Page 1996). To represent relationships among *DcSto*1 elements amplified from different sources, NJ tree was generated using MEGA 5.05 (Tamura et al. 2011). Consensus sequences of *DcSto*1 to *DcSto*9 were used to predict secondary structures in RNAfold (Hofacker 2003), to search for putative promoter regions using TSSP (Softberry), 3′-end cleavage and polyadenylation sites using POLYAH (Softberry), regulatory DNA sequences in RegSite database using NSITE-PL (Softberry), and to identify transposons inserted in/close to coding regions in the sequences deposited in GenBank using blastn algorithm (Altschul et al. 1997).

### Fluorescence in situ hybridization

Localization of *DcSto*1 elements on chromosomes of cv. Amsterdam 3 (AS33) was carried out by means of fluorescence in situ hybridization (FISH). The *DcSto*1 probe was amplified with DcS-TIR primer and cloned into pGEM-T vector. All steps of multi color FISH were carried out as described by Nowicka et al. (2012).

### Southern hybridization

Digestion of 10 μg genomic DNA was performed in 400 μl with 100 U of *EcoR*I (Fermentas) in 1× of *EcoR*I buffer for 3 h at 37 °C followed by 20 min at 65 °C. Digested DNAs were separated in 1 % TAE agarose gel for 16 h at 1 V/cm. The gel was washed for 8 min with 0.25 M HCl, twice for 15 min with denaturation buffer (0.5 M NaOH, 1.5 M NaCl), 1 min with distilled water, twice for 15 min with neutralization buffer (0.5 M Tris–HCl, pH 7.5, 1.5 M NaCl). After washing with 20× SSC (3 M NaCl, 0.3 M sodium citrate, pH7) for 15 min, the DNA was transferred onto Immobilion™-P transfer membrane (Millipore) at room temperature. The membrane was then exposed to UV light (120 mJ/cm^2^) using CL-100 Ultraviolet Crosslinker (UVP), washed for 2 min with 2× SSC, and dried. To prepare the probe, PCR was set up in 30 μl using 15 ng of template (a *DcSto*1 element cloned into pGEM-T) with 1 mM DcS-TIR primer, 0.1 mM digoxigenin-11-dUTP alkali-labile (Roche), 0.25 mM dNTPs (Fermentas), 1.5 U Long PCR Enzyme Mix (Fermentas) and 1× long PCR buffer. The temperature profile was as follows: 94 °C (1 min), 30 cycles of 94 °C (30 s), 50 °C (40 s), 68 °C (1 min) and 68 °C (4 min). Overnight hybridization at 65 °C was performed in 10 ml of hybridization buffer (7 % SDS, 50 % deionized formamide, 5× SSC, 0.1 % N-lauroyl sarcosine, 2 % blocking solution, 50 mM sodium phosphate, pH 7.0) with 30 μl of the denatured probe. Detection was carried out using DIG luminescent detection kit (Roche) following instructions provided by the manufacturer.

## Results

### Identification and characteristics of *DcSto* elements

**Fig. 1:**
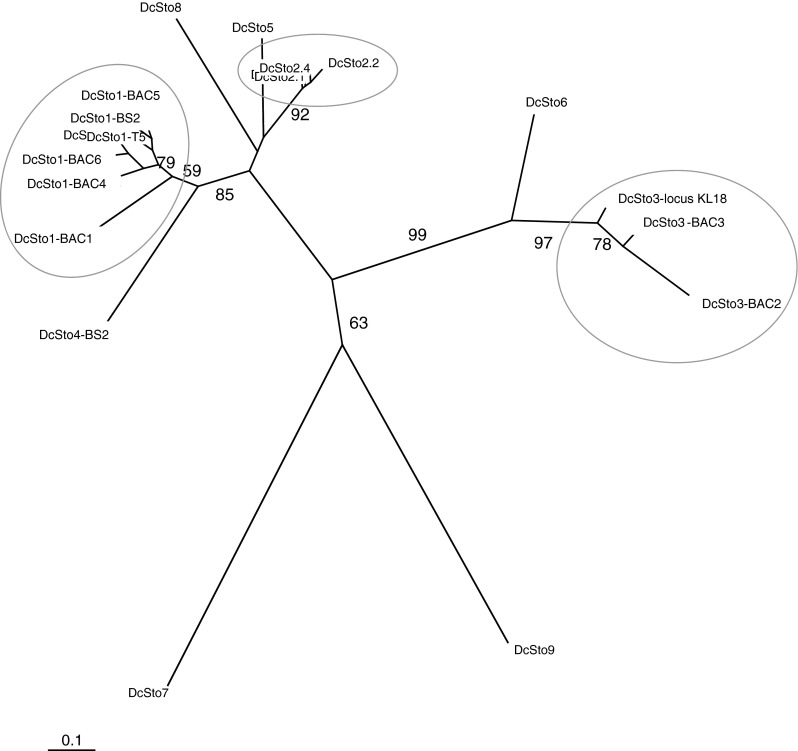
A Neighbor-Joining tree showing relationships among carrot *DcSto* elements. Clusters comprising copies of *DcSto*1, *DcSto*2, and *DcSto*3 elements are *circled*

Upon a routine analysis of length polymorphism at the *rs* locus (Yau et al. 2005) in carrot breeding materials of American origin we identified an undescribed 274 bp-long insertion characterized by a two-nucleotide ‘TA’ TSD and 16 bp-long TIRs. Based on the presence of TSD and TIR sequences, the inserted element was classified as a *Stowaway*-like MITE and named *DcSto*1-*rs*. Sequence of that element served as a starting point to identify more copies of *DcSto*. In total, we identified 89 elements using different methods (Table 1). On the basis of the commonly accepted 80-80-80 criterion (Wicker et al. 2007), the *DcSto* elements were divided into nine families, *DcSto*1 to *DcSto*9. Grouping of twenty *DcSto* for which the full sequence was available (i.e. excluding those identified by amplification with the DcS-TIR primer) is in Fig. 1. Members of *DcSto*1, *DcSto*2, and *DcSto*3, were represented by more than one element in each category. Elements *DcSto*4, *DcSto*5, and *DcSto*6 were related to *DcSto*1, *DcSto*2, and *DcSto*3, respectively while *DcSto*7, *DcSto*8, and *DcSto*9 were more distant.

**Table 1:** Number of *DcSto* copies and methods used for their identification

MITE family	The method used for identification					
	TIR-PCR^a^ from genomic DNA	TIR-PCR^a^ from BAC clones	iPCR	BES in silico analysis	Site-specific PCR	Reanalysis of published sequences
*DcSto*1	62	3	2	5	–	–
*DcSto*2	–	1^b^	1	1	1	–
*DcSto*3	2	1	–	2	1	–
*DcSto*4	–	–	–	–	1	–
*DcSto*5	–	–	–	–	1	–
*DcSto*6	–	–	–	–	1	–
*DcSto*7	–	–	–	–	–	1 (*AOX2a*, GQ248714, Cardoso et al. 2009)
*DcSto*8	–	–	–	–	–	1 (*gDcPAL3*, AB089813, Kimura et al. 2008)
*DcSto*9	–	–	–	–	–	1 (lipid body membrane protein, S47635, Hatzopoulos et al. 1990)

^a^Amplification with DcS-TIR primer specific to TIRs of *DcSto*1

^b^Identified as nested insterion in *DcSto*1, see Fig. 1b

**Fig. 2:**
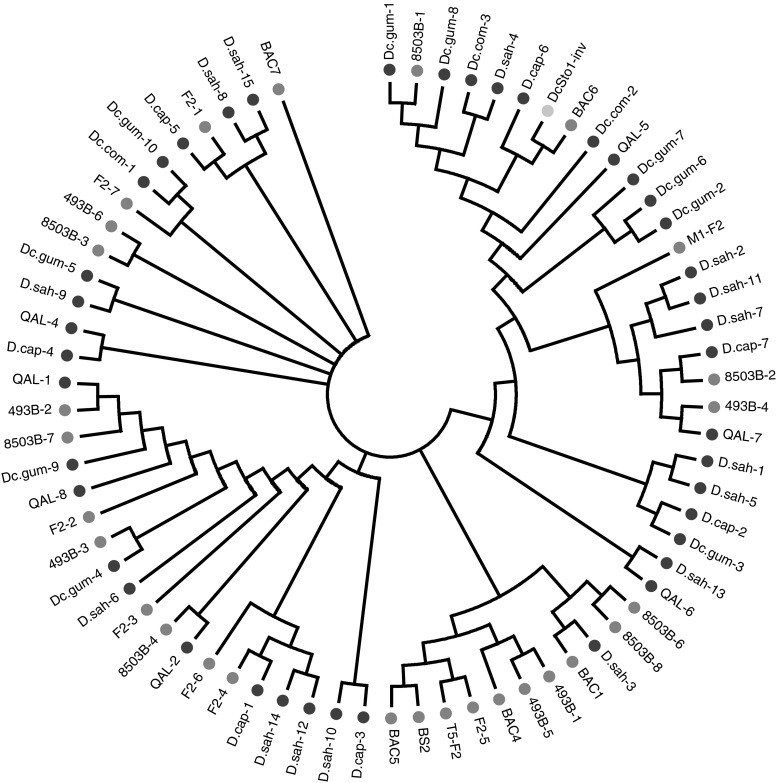
A Neighbor-Joining tree showing relationships among *DcSto*1 elements originating from different *Daucus* host genomes. *DcSto*1-*rs* is *labeled red*, *DcSto* elements amplified from cultivated carrot are *labeled green* and those from wild *Daucus* (sub)species are *labeled blue*. (Color figure online)

Among the *DcSto* elements identified, 73 copies represented *DcSto*1 and their average sequence similarity was 86 %. They could be grouped into branches on the Neighbor-Joining tree, however, the grouping did not correspond with their origin from cultivated versus wild *Daucus* (Fig. 2). For *DcSto*2 and *DcSto*3, four and six copies, respectively, were observed and each of the remaining six families was represented by one element.

**Fig. 3:**
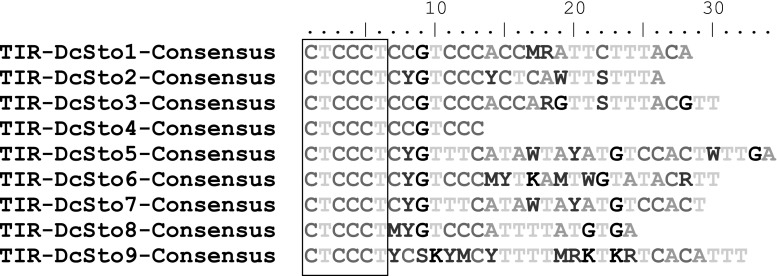
Alignment of TIR sequences of *DcSto* families (Y stands for T/C, W for A/T, S for C/G, R for A/G and M for A/C). The *red rectangle* indicates the conserved 6 nt-long TIR portion. (Color figure online)

The TIR length varied from 13 bp for *DcSto*4 to complete sequence folding into almost perfect hairpin structures in *DcSto*3, *DcSto*6, and *DcSto*9 (supplementary Figure 1). However, a highly conserved 6 bp-long terminal motif CTCCCT was always distinguishable (Fig. 3). As observed for other MITEs, the AT content of *DcSto* sequences was high (60–72 %).

### Abundance and genomic distribution of *DcSto*1

**Fig. 4:**
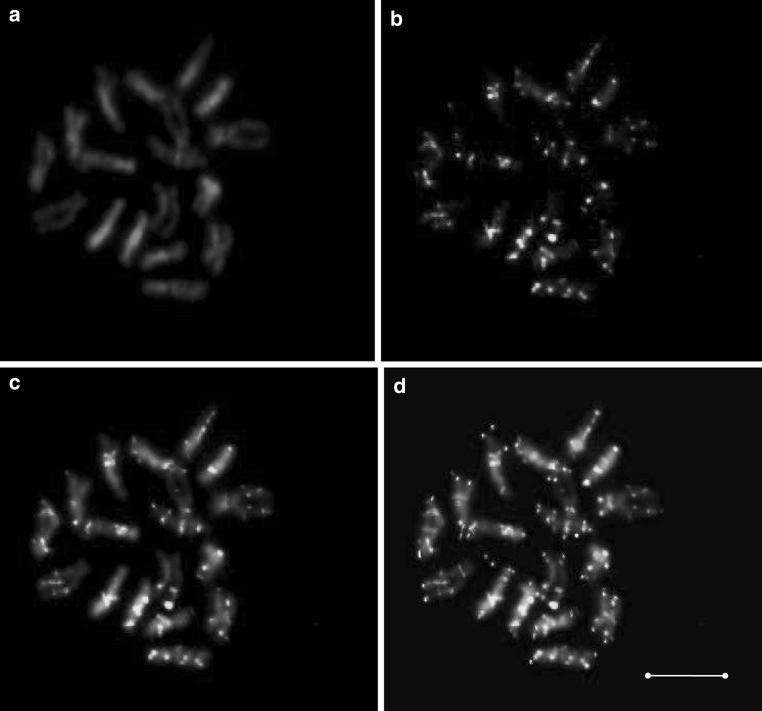
Localization of *DcSto*1 elements along carrot cv. Amsterdam 3 (AS33) chromosomes by multi-color fluorescence in situ hybridization (**a**–**d**). DAPI-stained mitotic chromosomes (*blue*) (**a**); distribution of the *DcSto*1 probe detected with FITC-conjugated anti-digoxigenin antibody (*green*) (**b**); distribution of the *DcSto*1 probe detected with FITC-conjugated anti-digoxigenin antibody (*green*) on DAPI-stained chromosomes (*blue*) (**c**); overlapped hybridization signals of the *DcSto*1 probe (*green*), the centromeric probe [Cy5, *red* signal was changed to purple with Case Data Manager (ASI)] and the telomeric probe [Cy3, *red* signal was changed to yellow with Case Data Manager (ASI)] on the same metaphase stained with DAPI (*blue*) (**d**). *Scale bar* = 5 μm. (Color figure online)

PCR amplification with the DcS-TIR primer was set up to screen 141 randomly chosen BAC clones. Amplicons of expected size were present in 87 of them. Thus, on average one *DcSto*1 element was present per 196 kb and the copy number of *DcSto*1 could be estimated as ca. 5,000 per diploid carrot genome. The high copy number of *DcSto1* elements was confirmed by Southern hybridization with the *DcSto*1 element used as a probe. Hybridization to *EcoR*I-digested DNA of cultivated and wild carrots resulted in a smear, indicating presence of *DcSto*1 in numerous copies (supplementary Figure 2). We also investigated the physical distribution of *DcSto*1 along the carrot chromosomes using fluorescent in situ hybridization. *DcSto*1 elements were present on both arms of all chromosomes but they were absent in the centromeric, telomeric, subtelomeric, and nucleolar organizer regions. In general, *DcSto*1 showed clustered distribution along the euchromatic regions of chromosome arms and their co-localization with the DAPI stained blocks of heterochromatin was not observed (Fig. 4).

### Local rearrangements resulting from the activity of *DcSto* elements

**Fig. 5:**
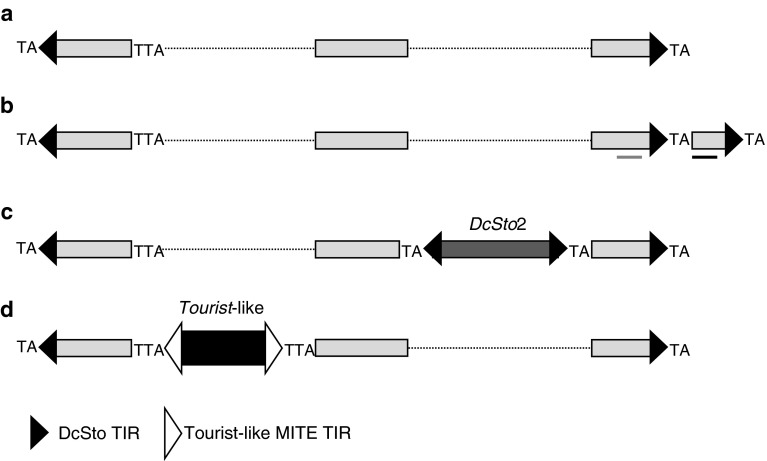
Graphic representation of nested insertions identified in PCR fragments generated with DcS-TIR primer anchored in *DcSto*1 TIRs. A complete *DcSto*1 sequence (**a**), a duplicated terminal *DcSto* sequence, *red line* indicates the region of *DcSto* with 93 % similarity to *DcSto*-*rs* and *blue line* indicates the region of *DcSto* with 73 % similarity to *DcSto*-*rs* (**b**), nested insertion of *DcSto*2 (**c**), nested insertion of a *Tourist*-like MITE (**d**), not drawn to scale. Target site duplications are marked as TA (*Stowaway* type) and TTA (*Tourist* type), *dotted lines* represent gaps, *yellow bars* represent *DcSto*1, *orange* and *green bars* represent nested insertions of *Tourist*-like MITE and *DcSto*2, respectively. (Color figure online)

PCR amplification of *DcSto* transposons from carrot BAC clones using DcS-TIR primer produced three bands of sizes longer than expected for *DcSto*1. One of them was a *DcSto*1 element carrying additional 59 nt similar to the terminal part of the transposon at the 5′ end (Fig. 5b). Those extra nucleotides, excluding the sequence of the TIR-primer, were more similar to other *DcSto* copies then to the adjacent element, suggesting a nested insertion into another *DcSto* element. Two other products were derived from *DcSto*1 carrying nested insertions of a complete *DcSto*2 element or an unrelated *Tourist*-like MITE (Fig. 5c, d).

To characterize local variation in *DcSto* insertion sites, primers flanking *DcSto* insertions identified in BES were used to re-amplify eight loci in unrelated individuals. All insertion sites were polymorphic among analyzed plants of cultivated carrot.

**Fig. 6:**
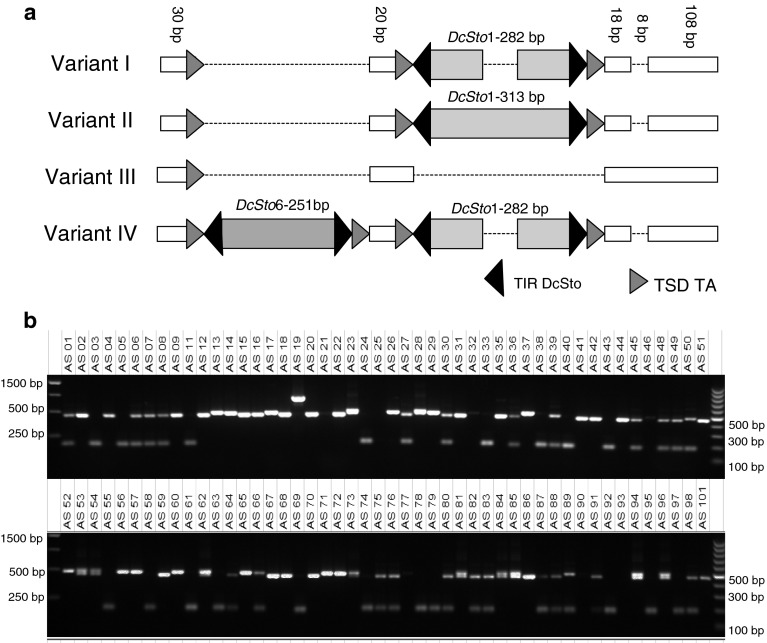
Schematic representation of structural variants identified in BS2.1 locus (**a**) and amplification profiles for a collection of plants representing diversity of the cultivated carrot (**b**). *Yellow* and *red bars* indicate insertions of *DcSto*1 and *DcSto*6 elements, respectively. (Color figure online)

In case of three loci, besides expected size variants representing empty/occupied site, more complex rearrangements were also observed. In the BS2.1 region, a complete *DcSto*1 was identified, but also two of its derivatives, likely resulting from abortive gap repair following an excision event. Additionally, one of the derived *DcSto*1 variants was accompanied by an insertion of a *DcSto*6 element 20 bp upstream the *DcSto*1 insertion (Fig. 6). We have not identified a variant carrying a solo insertion of *DcSto6*, which suggests that the latter element was inserted into the BS2.1 variant with the internally truncated *DcSto*1 element already present. The BS2.2 region was characterized by alternative insertions of two different *DcSto* elements, *DcSto*3 and *DcSto*4, in exactly the same position. Also, a short deletion around the insertion site, likely resulting from an excision event, was observed in one of the variants (supplementary Figure 3). In the BS4 region, three independent insertions of *DcSto*1, *DcSto*5, and an uncharacterized 549 bp-long indel were found in different genetic backgrounds (supplementary Figure 4). *DcSto*5 was inserted 48 bp upstream the *DcSto*1 insertion site and a variant carrying insertions of both elements was not identified.

The presence of non-fixed *DcSto* insertions in *Daucus carota* suggests an extensive recent transpositional activity of those elements. One particularly interesting case of *DcSto* mobilization came from an analysis of size polymorphism in the first intron of the carrot *chxb1* gene. A longer version of the intron, resulting from the insertion of a *DcSto*2 element was present and segregating only in AS38, one of more than 160 screened accessions (supplementary Figure 5), indicating a very recent insertion event limited to a single population of cultivated carrots.

### Presence of *DcSto* elements in the vicinity of coding regions

*DcSto* elements were identified upon examination of published sequences in the vicinity of genes of *Apioideae* (carrot, *Petroselinum crispum*, and *Bupleurum kaoi*), especially within 5′ UTR regions and upstream of transcription start sites (Table 2). Complete elements were identified upstream of two carrot genes, i.e. *gDcPAL3*, and Inv*Dc5. Interestingly, other *DcSto* copies associated with genic regions were devoid of TIR and the typical TSD at one or both ends, thus they were immobilized.

**Table 2:** Regions carrying insertions of *DcSto*-like elements close to or in coding sequences of *Apioideae*

Accession number	Gene	*DcSto* characteristics	Localization
*DcSto*1			
AB050962	*Daucus carota* *DcECP40* gene for ECP40, promoter region	Lack of two TIRs	Promoter region
AJ303199	*Daucus carota* *sut2* gene for sucrose/proton symporter, exons 1–4	Lack of one TIR	Intron
*DcSto*3			
AB118876	*Daucus carota* *C*-*ABI3* gene, upstream region	Lack of one TIR	Promoter region
*DcSto*5			
AB089813	*Daucus carota* *gDcPAL3* gene for phenylalanine ammonia-lyase	Complete	1,933 bp upstream of cds
*DcSto*6			
X16131	*D. carota* *DC8* gene for an embryonic-specific 66 kDa protein	Lack of one TIR	761 bp upstream of cds
U46217	*Petroselinum crispum* common plant regulatory factor *CPRF1* gene, promoter region and complete cds	Complete	Intron
X55736	*Petroselinum crispum* *PR2* gene for pathogenesis-related protein 2	Complete	Promoter region
AF274564	*Petroselinum crispum* immediate-early fungal elicitor protein *CMPG1* gene, partial cds	Complete	1,391 bp upstream of cds
AF121354	*Petroselinum crispum* transcription factor *WRKY3* gene, partial cds	Complete	3,417 bp upstream of cds
AF239835	*Petroselinum crispum* fatty acid desaturase/hydroxylase-like protein *ELI7.1* gene, complete cds; and fatty acid desaturase/hydroxylase-like protein *ELI7.2* gene, partial	Partial	Between two predicted cds (ELI7.1 and ELI7.2)
Y16091	*Daucus carota* Susy*Dc2 gene	Lack of one TIR	3′ UTR
Y18706	*Daucus carota vacuolar invertase isoenzyme II* gene (Inv*Dc5)	Complete	5′ UTR
*DcSto*7			
AB188289	*Daucus carota C*-*EF1* mRNA for embryonic element binding Factor 1, partial cds	Partial	3′ UTR
*DcSto*8			
FN667831	*Bupleurum kaoi* mRNA for ethylene response factor 1 (*erf1* gene)	Partial	3′ UTR

*DcSto6* showed 70 % similarity over the entire element to the complete *Stowaway*-like MITE in parsley, which we named *PcSto* (*Petroselinum crispum Stowaway*), identified in a region upstream of four *P. crispum* genes and in an intron of one gene (Table 2). An average sequence similarity of the four identified *PcSto* elements was 74 %, and only *PcSto*-PR2 and *PcSto*-CMPG1 were over 80 % similar. Interestingly, *DcSto*6 and *DcSto*3 were more similar to the *PcSto* elements than to other carrot *DcSto* elements (supplementary Figure 6).

*DcSto* elements belonging to five families carried putative promoters and TATA boxes, while four families might provide polyA sites for adjacent genes. Also, within sequences of any *DcSto* family at least 5 putative regulatory motifs were present and only three families i.e. *DcSto*5, *DcSto*7, and *DcSto*8 carried less then ten putative regulatory motifs (Table 3).

**Table 3:** Coordinates of putative promoters, regulatory motifs, and polyA signals within consensus *DcSto* sequences

Family	Position/strand of the first nt of predicted promoters	Number/strand of predicted transcription factor binding sites	Number/strand of regulatory motifs	Number/position/strand of predicted polyA signals
*DcSto*1	189/+	39/12(+), 27(−)	21/13(+), 8(−)	0
*DcSto*2	167/−	15/7(+), 8(−)	12/8(+), 4(−)	1/137/−
*DcSto*3	–	0	10/5(+), 5(−)	1/104/+
*DcSto*4	130/+	12/4(+), 8(−)	12/4(+), 8(−)	0
*DcSto*5	–	0	5/3(+), 2(−)	0
*DcSto*6	142/+	30/12(+), 18(−)	14/7(+), 7(−)	1/112/+
*DcSto*7	–	0	5/2(+), 3(−)	0
*DcSto*8	227/+	32/8(+), 24(−)	7/1(+), 6(−)	2/141,164/+, −
*DcSto*9	–	0	16/5(+), 11(−)	0

## Discussion

The present study demonstrated that *DcSto* MITEs were abundant and diverse in the carrot genome. We investigated the distribution of *DcSto*1 elements across cultivated and wild carrot, as well as two closely related species *D. capillifolius* and *D. sahariensis*. We did not observe any *DcSto*1 lineages differentiating investigated groups of accessions, which might reflect their previously documented intra- and interspecific crossability (Grzebelus et al. 2011). We showed that *DcSto* elements in carrot were present in thousands of copies, which stays in agreement with the general characteristics of *Stowaway* elements present in other plants. For example, 18 *Stowaway*-like MITE families present in over 18,000 copies, were described in the wheat genome (Yaakov et al. 2013). Also, analysis of *Stowawa*y elements in the relatively small genome of rice revealed presence of over 22,000 *Stowaway* elements divided into 36 families (Feschotte et al. 2003).

The high level of *DcSto* insertion polymorphism and low frequency of carrot plants harboring insertions of *DcSto*1 into *rs* and *DcSto*2 into *chxb1* genes suggests recent mobilization of *DcSto* elements. Insertion and excision events resulted in high local variability, including deletions of sequences surrounding the excision site, which was reported previously in rice (Yang et al. 2006, 2009).

We speculate that the *DcSto*3 and *DcSto*6 families were vertically inherited from the common ancestor by the *Daucus* and *Petroselinum* linkages as suggested by their high similarity to *PcSto* identified in the *P. crispum* genome. Despite the apparent long evolutionary history of these families, indicated by their likely presence in the genome of a common ancestor of *Daucus* and *Petroselinum*, a recent mobilization event of *DcSto*6 in the BS2.1 region was documented. As *Stowaway* MITEs were shown to be evolutionarily related to autonomous elements from the *Tc1/mariner* superfamily (Turcotte et al. 2001; Menzel et al. 2006), it has been commonly accepted that their mobilization relies on the availability of *mariner* transposases (Feschotte et al. 2003). However, autonomous elements serving as transposase donors for *DcSto* elements remain to be identified. As proposed by Jiang et al. (2004), MITE precursors originated from autonomous elements, but their proliferation was driven later by transposases encoded by autonomous elements, not directly related but still capable of recognizing MITE termini. Also, relatively few changes in TIRs may have a dramatic effect on transposase binding to the element ends (Lampe et al. 2001). According to this scenario, divergence of TIRs sequences of *DcSto* elements may have resulted in mobilization of a particular group by different *mariner*-like transposases within overlapping time-frames. A hypothesis of multiple bursts of transposition was also proposed by Grzebelus et al. (2009) to explain diversity and evolutionary history of *Medicago* PIF/*Harbinger*-related MITEs.

We found evidence for clustered insertions of *DcSto*. FISH with *DcSto*1 revealed clustered signals over all carrot chromosomes. Analysis of the local structure of carrot *DcSto* insertion sites revealed clustered insertions (BS2.1) and independent insertions of *DcSto* into the same position or very close to the insertion site of other elements in plants of different origin (BS2.2, BS4). Analysis of maize and rice MITEs insertion site preference shows that insertion of MITEs into other members of the same family were common. It was proposed that nested and clustered insertions may act as a mechanism of limitation of transposition frequency and ‘safe haven’ where further integration of transposons would be tolerated (Rothnie et al. 1990; Jiang and Wessler 2001).

Independent insertions of *Stowaway* elements into the same localization were shown in rice R genes (Hu et al. 2000) and *Triticaeae* β-amylase gene (Mason-Gamer 2007). In addition, *Stowaway*s were identified in maize *bz* locus, referred to as a transpositional ‘hot spot’ and characterized by multiple insertions of MITEs, DNA transposons, and retrotransposons (Wang and Dooner 2006). The first *DcSto* element was found in the first intron of carrot soluble invertase isozyme II (*rs*). Interestingly, the same intron was previously reported as harboring insertion of a non-autonomous *PIF/Harbinger* element *DcMaster*1 (Grzebelus et al. 2006) which might suggest that the intron acted as a similar transpositional ‘hot spot’.

As observed for other MITEs, *DcSto* elements were frequently inserted in the vicinity of genes (Mao et al. 2000, Yaakov et al. 2013). Besides the *rs* gene, we found a copy of *DcSto* in the first intron of the *chxb1* gene and reanalyzed previous reports on insertions in or near carrot genes. Cardoso et al. (2009) identified an indel in the third intron of the *AOX2a* gene which we found to be a *DcSto*. Kimura et al. (2008) described an insertion of a 299 bp MITE in the promoter region of the phenylalanine ammonia-lyase gene (*DcPAL*3) close to another MITE element. The former MITE could be attributed to the *DcSto* group. Also, we identified a *DcSto* element inserted in the region upstream carrot lipid body membrane protein gene (Hatzopoulos et al. 1990).

Presence of putative promoters and polyA motifs within sequence of most of analyzed *DcSto* elements and presence of sequences constituting putative regulatory elements for all families might indicate their possible influence on the expression of adjacent genes. The effect of *DcSto*8 on the expression of carrot phenylalanine ammonia-lyase gene (*DcPAL3*) analyzed by Kimura et al. (2008), showed that clustered insertion of *DcSto*8 and another MITE significantly increased the transcription level. That effect may be one of the reasons for a relatively frequent occurrence of *DcSto* elements in clusters with other MITEs observed previously and confirmed in the present study. Analysis of the binding capacity of a region upstream a lipid body membrane protein gene from carrot overlapping with the *DcSto*9 insertion showed that the region had a potential to form complexes with nuclear extracts from embryos (Hatzopoulos et al. 1990). Interestingly, the region identified as responsible for DNA-binding, harbored by *DcSto*9, was not present in other *DcSto* element families.

Very recently, it was shown that more than half of miRNAs associated with rice transposable elements originated from MITEs (Yu et al. 2010, Sanan-Mishra et al. 2009). Similar results were observed in *Solanaceae* (Kuang et al. 2009) and *Arabidopsis* (Hollister et al. 2011). Read-through transcription of MITEs inserted into UTR regions of genes may lead to their folding into hairpin structures, which are further processed into small RNAs. As shown in *Arabidopsis*, such MITE-derived miRNAs have a significant impact on the decrease of expression of adjacent genes, owing to higher methylation of those regions (Hollister et al. 2011). A predicted miRNA encoded by *DcSto*7, as proposed by Cardoso et al. (2009), showed significant similarity to a rice miRNA. Moreover, *DcSto*3 and *DcSto*6 copies, capable of forming fold-back structures, were most frequently associated with carrot transcripts (Iorizzo et al. 2011). Also, *DcSto*6 lacking one of the TIRs is present in the region upstream the carrot *Dc8* gene showing differential expression during embryo development related to changes in the methylation pattern of the promoter region (Zhou et al. 1998). Also, *DcSto*6 elements shared 69 % similarity over their entire sequence with *PcSto* elements, identified adjacent to coding regions of *Petroselinum crispum*, a related *Apiaceae* species. In addition, *DcSto* and related elements in *Apioideae* species identified in the vicinity of coding regions were frequently characterized by the lack of one or both TIRs. Loss of functional termini prevents mobilization of *DcSto* elements which might suggest their retention due to a putative adaptive effect on the expression of adjacent genes. We conclude that abundance of *DcSto* elements in euchromatic regions, their presence in carrot transcripts, and presence of putative regulatory motifs within their sequences may indicate their involvement in the regulation of gene expression.

## Electronic supplementary material

Below is the link to the electronic supplementary material.

Supplementary material 1 (PDF 646 kb)
