# Supplementary material for: DcSto: carrot Stowaway-like elements are abundant, diverse, and polymorphic
Source: Genetica. 2013 Jun 18;141(4):255–67. doi: 10.1007/s10709-013-9725-6 (PMC3695323; doi:10.1007/s10709-013-9725-6)
Supplement: Supplementary file 1 — Supplementary material 1 (PDF 646 kb) [file 10709_2013_9725_MOESM1_ESM.pdf]

***DcSto* – carrot *Stowaway*-like elements are abundant, diverse, and polymorphic**

Genetica

Alicja Macko-Podgorni[1], Anna Nowicka[1], Ewa Grzebelus[1], Philipp W. Simon[2], Dariusz Grzebelus[1]

[1] Department of Genetics, Plant Breeding and Seed Science, University of Agriculture in Krakow, Al. 29 Listopada 54, 31-425 Krakow, Poland

[2] USDA-ARS Vegetable Crops Research Unit and Department of Horticulture, University of Wisconsin-Madison, 1575 Linden Drive, Madison, WI 53706, USA

e-mail address of the corresponding author: **d.grzebelus@ogr.ur.krakow.pl**

supplementary materials

Supplemental Table 1. The list of plant materials.

Plant code	Source	Cultivar	Geographic origin
**AS 01**	Bejo	White Satin	Europe
**AS 02**	Bejo	Yellowstone	Europe
**AS 03**	Seminis	710015	USA
**AS 04**	Seminis	Nutrired	USA
**AS 05**	Seminis	Santa Cruz	USA
**AS 06**	Bejo	Nerac F1	Europe
**AS 07**	Bejo	Deep Purple F1	Europe
**AS 08**	Bejo	Purple Haze F1	Europe
**AS 09**	Seminis	Anthonina	USA
**AS 10**	JKI	Schweizer Rübli	Europe
**AS 11**	INH	Blanche 1/2 longue des vosges	Europe
**AS 12**	WGRU	Persia No. 242	Asia
**AS 13**	WGRU	Gelbe Rheinische	Europe
**AS 14**	DAU	landrace	Asia
**AS 15**	WGRU	Afghan Purple	USA
**AS 16**	DAU	Senta	Europe
**AS 17**	NGB	Nantes Fancy	Europe
**AS 18**	WGRU	Nagykallo	Europe
**AS 19**	WGRU	Himuro Fuyugosi Gosun No.2	Japan
**AS 20**	POLAN	Nantejska Polana	Europe
**AS 21**	MKS	Hakata Kintoki	Japan
**AS 22**	WGRU	Pusa Kesar	Asia
**AS 23**	WGRU	Panipat Special	Asia
**AS 24**	WGRU	Syrian Purple	Asia
**AS 25**	Wild	JKI-Selektion	Asia
**AS 26**	NGB	Nantes Apollo	Europe
**AS 27**	MKS	China Yellow	Asia
**AS 28**	WGRU	Shahpur Special	Asia
**AS 29**	MKS	Kokubu Senko Oonaga	Japan
**AS 30**	MKS	Sapporo Futo (Danvers)	Japan
**AS 31**	MKS	Shima Ninjin	Japan
**AS 32**	MKS	Hekinan Senko 5sun	Japan
**AS 33**	Commercial	Amsterdam 3	Europe
**AS 34**	BVRC	Seeds from Beijing	Asia
**AS 35**	WGRU	White Belgian	Europe
**AS 36**	Commercial	Lobbericher	Europe
**AS 37**	WGRU	Gajar	Asia
**AS 38**	WGRU	Mestnaya	Russia
**AS 39**	JKI	JKI-Selektion	Asia
**AS 40**	JKI	JKI-Selektion	Asia
**AS 41**	WGRU	Lozin147	Europe
**AS 42**	WGRU	Delta a Cuoro Rosso	Europe
**AS 43**	USDA	HCM	USA
**AS 44**	WGRU	Western Red	Australia
**AS 45**	WGRU	Norfolk Giant	Europe
**AS 46**	WGRU	Kuettiger	South America
**AS 47**	DAU	Yellow Belgian	Europe
**AS 48**	WGRU	Bitolski	Europe
**AS 49**	WGRU	Gajar	Asia
**AS 50**	WGRU	Mestnaya	Russia
**AS 51**	WGRU	Red Elephant	Europe
**AS 52**	WGRU	Victa	Europe
**AS 53**	WGRU	Niiza Etton Gosun	Japan
**AS 54**	Bejo	Nevis	Europe
**AS 55**	Sperli	Rotin	Europe
**AS 56**	Bejo	Vita Longa	Europe

Supplemental Table 1. continued.

Plant code	Source	Cultivar	Geographic origin
**AS 57**	NGB	Nantes Empire	Europe
**AS 58**	NGB	Touchon Format	Europe
**AS 59**	NGB	Amsterdamer Master	Europe
**AS 60**	DAU	Lange Rote Stumpfe ohne Herz 1	Europe
**AS 61**	NGB	Nana W 561	Europe
**AS 62**	WGRU	Shinsuu Senkou Oonaga	Japan
**AS 63**	WGRU	Benifuku Fuyngosi 5 Sun	Japan
**AS 64**	WGRU	Stratova	Europe
**AS 65**	DAU	Nantes Half Long	Europe
**AS 66**	WGRU	Champion Scarlet Horn	Europe
**AS 67**	WGRU	Viking	Europe
**AS 68**	WGRU	Berlicum Normaal	Europe
**AS 69**	WGRU	Purple Stem Selektion	Europe
**AS 70**	DAU	Danver's Red Core	Europe
**AS 71**	NGB	Nantes Duke	Europe
**AS 72**	WGRU	Red Giant	Europe
**AS 73**	Vilmorin	Bolero	Europe
**AS 74**	Vilmorin	Texto	Europe
**AS 75**	Vilmorin	Presto	Europe
**AS 76**	USDA	Beta III	USA
**AS 77**	JKI	Pariser Markt	Europe
**AS 78**	JKI	Vitaminaja	Russia
**AS 79**	WGRU	Cyrano	Europe
**AS 80**	DAU	Gold Pak	Europe
**AS 81**	Bejo	Rainbow F1	Europe
**AS 82**	Bejo	Mello Yello F1	Europe
**AS 83**	WGRU	St. Valery	Europe
**AS 84**	WGRU	Sytan	Europe
**AS 85**	NGB	Nantes Liva	Europe
**AS 86**	NGB	Flakkeer Regina	Europe
**AS 87**	WGRU	Long Red	Africa
**AS 88**	WGRU	Winter Perfection	Europe
**AS 89**	WGRU	Stupicka k Rychleni	Europe
**AS 90**	WGRU	Short n'Sweet	USA
**AS 91**	WGRU	Tropical	South America
**AS 92**	WGRU	Moskovskaja Zimniaja	Russia
**AS 93**	WGRU	Nyiregyhaza	Europe
**AS 94**	WGRU	Yamanouchi Ishyaku Senko	Japa
**AS 95**	NGB	London Torve,<br>B Tagenshus III	Europe
**AS 96**	WGRU	Berlicumer<br>Bercoro RS	Europe
**AS 97**	NGB	Nantes Palisade	Europe
**AS 98**	WGRU	Berlikum Perfecta	Europe
**AS 101**	WGRU	Wild, *Daucus aureus*	Asia

Bejo – Bejo Zaden, BVRC-Beijing Vegetable Research Center, Beijing, China; DAU – Leibniz Institute of Plant Genetics and Crop Plant Research; INH – Institut National d'Horticulture et de Paysage, Angers, France; JKI – Julius Kuehn Institute, Quedlinburg, Germany; MKS – Mikado Kyowa Seed Co. Ltd., Chosei, Japan; NGB – Nordic Genetic Resource Center, Alnarp, Sweden; POLAN – Krakowska Hodowla i Nasiennictwo Ogrodnicze POLAN Ltd., Krakow, Poland; Seminis – Seminis Vegetable Seeds, Inc., Saint Louis, USA; Sperli – Sperli – Saatzucht Carl Sperling & Co. GmbH, Lüneburg, Germany; USDA – USDA-ARS, University of Wisconsin, Department of Horticulture, Wisconsin, USA; Vilmorin – Vilmorin & Cie Co., La Ménitré, France; WGRU – Warwick Genetic Resources Unit, Warwick University, Wellesbourne, Great Britain; Wild – Rudolf Wild GmbH & Co. KG, Berlin; commercial – seeds purchased from a retailer

Supplemental Table 2. The list of site-specific primers flanking *DcSto* insertions.

name	sequence (5’- 3’)	annealing temperature	PCR elongation time
*DcSto*1, flanking sequence obtained using iPCR			
SiT-1-F	TCDAUTCAAAATTCATTAGAACAA		
SiT-1-R	TGTTTAAGACCCTTGGCAAAA		
SiT-2-F	CGTGCCATATATTTTCTCGTCT	56^0C	1 min
SiT-2-R	AGAAAAAGTCAAGCGACGACT	56^0C	1 min
SiT-3-F [a]	AAGTTATAGTATAAATAAGCCAAATT	-	-
SiT-3-R [a]	CTCCTTCATGTCCACCACCT	-	-
SiT-4-F [a]	TCCATACACGTGTCGTTATCTT	-	-
SiT-4-R [a]	CTGGGAAACAGTGTGTTGGTT	-	-
SiT-5-F	TCGGTTGGTTTGTACTTTGG	55^0C	1 min
SiT-5-R	TAAACACATGCGAAGCACAT	55^0C	1 min
SiT-6-F	CGACGCAGCAAATTTCGTA	55^0C	1 min
SiT-6-R	TGACTGCATATGGCAATTTTGT	55^0C	1 min
SiM-1-F	TTCGCCTTTTGTGTAGCTGA	55^0C	1 min
SiM-1-R	TATDAUTGGCTGGAGTGGTG	55^0C	1 min
SiM-2-F	CCGGTGTTTGTCTCGTGTAA	60^0C	1 min
SiM-2-R	AGGTTTTGCCAGAAGCTGAA	60^0C	1 min
SiEc-1-F [a]	TTGTTGGCGGTAAGTCTCATAA	-	-
SiEc-1-R [a]	GGDAUAAAATTGCACTCCAAC	-	-
SiEc-2-F [a]	TCTGGTAGCGAAAGAGCAAAA	-	-
SiEc-2-R [a]	TGTTTGGTTGGGAAGTAGGG	-	-
SiN-1-F	CCCTCATAAATTTCTCGTCCA	55^0C	1 min
SiN-1-R	TCAADAUAGAAAAGGGCAGTCA	55^0C	1 min
SiN-2-F [a]	TCAATCATGCCTCCAGTTCA	-	-
SiN-2-R [a]	TGCAGTATTGTTCTTCGTGTAAAA	-	-
SiM-3-F	GCAGGTAGAATCTTTTAGGAAACTC	55^0C	1 min
SiM-3-R	CCGACTTCAAAGTTTCCCTTT	55^0C	1 min
SiN-3-F	AAAAAGAAGCCAACTTCTCGTG	55^0C	1 min
SiN-3-R	TTCGADAUGGACCCATATATCC	55^0C	1 min
*DcSto*1, flanking sequence obtained using BES *in silico* analysis			
DcS-BAC1-F	TCCTCCGCATTTCACTTTTC	55^0C	1 min
DcS-BAC1-R	GGTDAUATAACCCACCACCA	55^0C	1 min
DcS-BAC2.1-F	TTAAGGCCATACCATTATACTTCA	55^0C	1 min
DcS-BAC2.2-F	TGGTTGAATATGTGTGGTTGTG	55^0C	1 min
DcS-BAC2.1-R	TCAGCAGCAACCACTTTGTC	55^0C	1 min
DcS-BAC2.2-R	TGAAGTATAATGGTATGGCCTTAA	55^0C	1 min
DcS-BAC3-F	GCCAATTTGTCCAAATCTCAA	55^0C	1 min
DcS-BAC3-R	GCGCGACCTATGCADAUTAT	55^0C	1 min
DcS-BAC4-F	GCCAAGGTGGACAAAATCAT	55^0C	1 min
DcS-BAC4-R	CGTTTCAAGTGGCAGDAUAATA	55^0C	1 min
DcS-BAC5-F	GGCCACAATCAGDAUAAGTTTC	55^0C	1 min
DcS-BAC5-R	TCGGGACTGTTCATAACATGAG	55^0C	1 min
DcS-BAC6-F	GGCTCGAGTGTTAGCCATGA	55^0C	1 min
DcS-BAC6-R	GAAATACGTCCTCCGTCCAA	55^0C	1 min
DcS-BAC7-F	GTTGCCTACCGAAGAAGGAAAG	55^0C	1 min
DcS-BAC7-1R	AGGGGTCCAAGGTGDAUATAGT	55^0C	1 min
DcS-BAC7-2R	TGTTGCATAAGAGCAAGTCCA	55^0C	1 min

[a] Primers for which no amplification was observed while PCR with annealing temperature ranging from 54 ^{0}C to 60^0C and elongation time from 1-6 min.

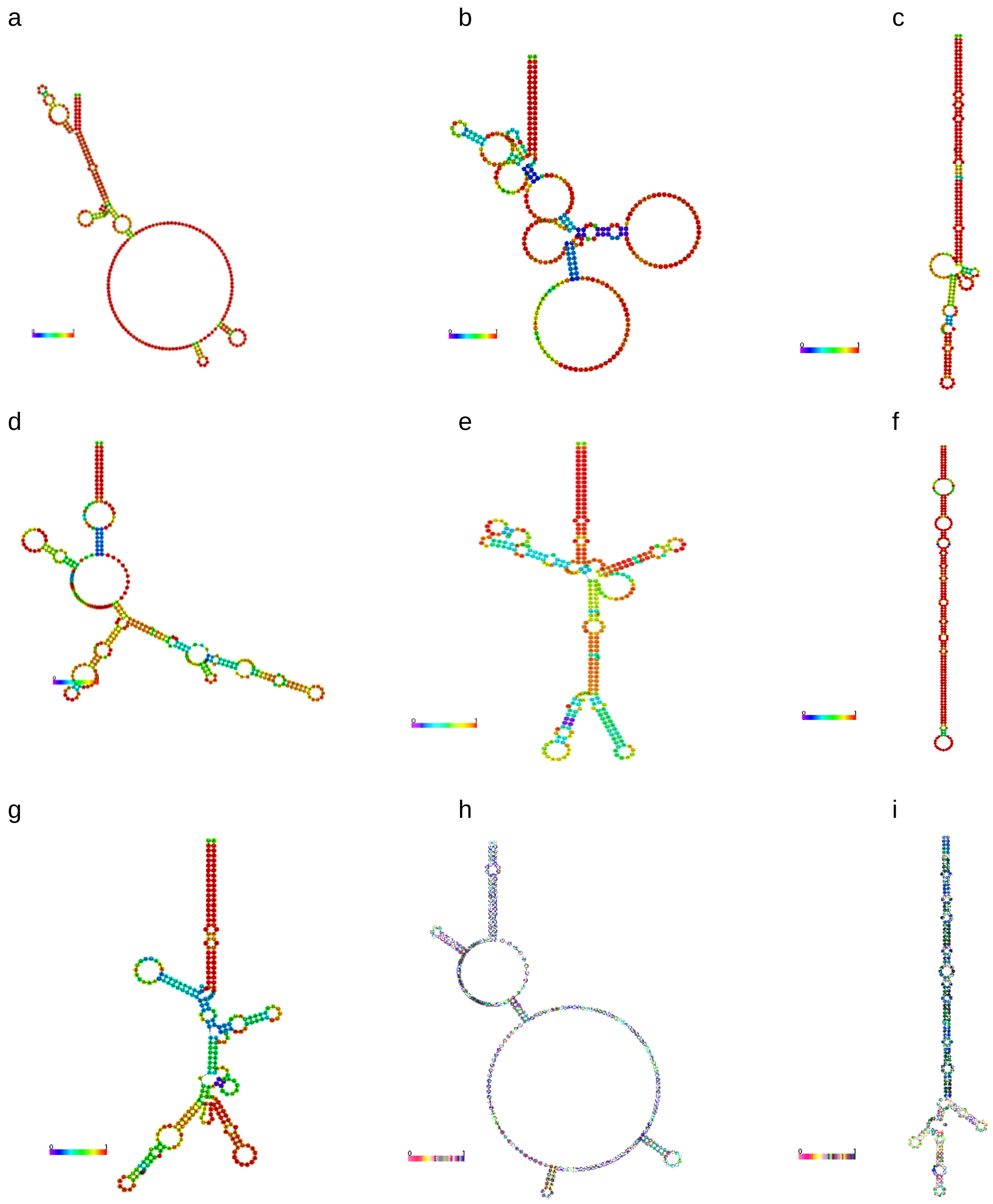

Supplemental Figure 1. Predicted MFE secondary structures of *DcSto*1 (**a**), *DcSto*2 (**b**), *DcSto*3 (**c**), *DcSto*4 (**d**), *DcSto*5 (**e**), *DcSto*6 (**f**), *DcSto*7 (**g**), *DcSto*8 (**h**), *DcSto*9 (**i**) created with RNAfold.

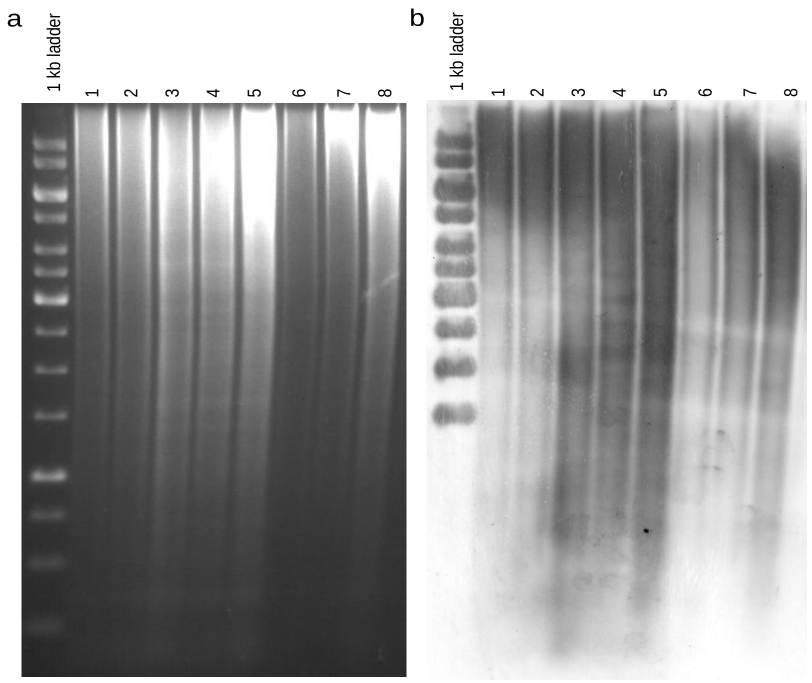

Supplemental Figure 2. Southern hybridization of a *DcSto*1 probe to the genomic DNA of *Daucus* digested with *EcoR*I. Agarose electrophoresis of restriction fragments (10 µg of DNA per lane) (**a**), the result of hybridization (**b**). Lanes: 1- *D. carota* subsp. *sativus* cv. Gold Pak, 2-*D. carota* subsp. *gummifer,* 3-*Daucus pusillus,* 4-*Daucus capillifolius,* 5- *D. carota* subsp. *sativus* cv. Brasilia, 6-*Daucus carota* subsp. *carota,* 7 *D. carota* subsp. *sativus,* Indian landrace, 8- *D. carota* subsp. *sativus* cv. Long Red.

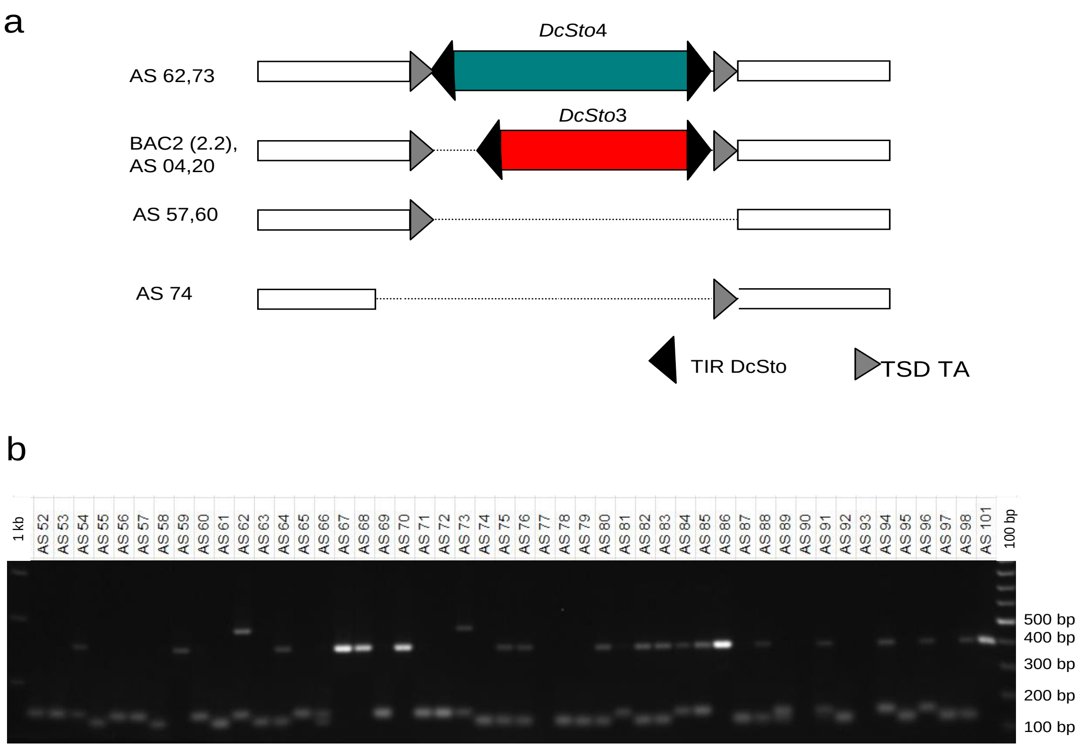

Supplemental Figure 3. A schematic representation of structural variants identified in BS2.2 locus (**a**), amplification profiles for a collection of plant representing the diversity of cultivated carrot (**b**). White box represents DNA flanking insertion site and dotted line represents gaps.

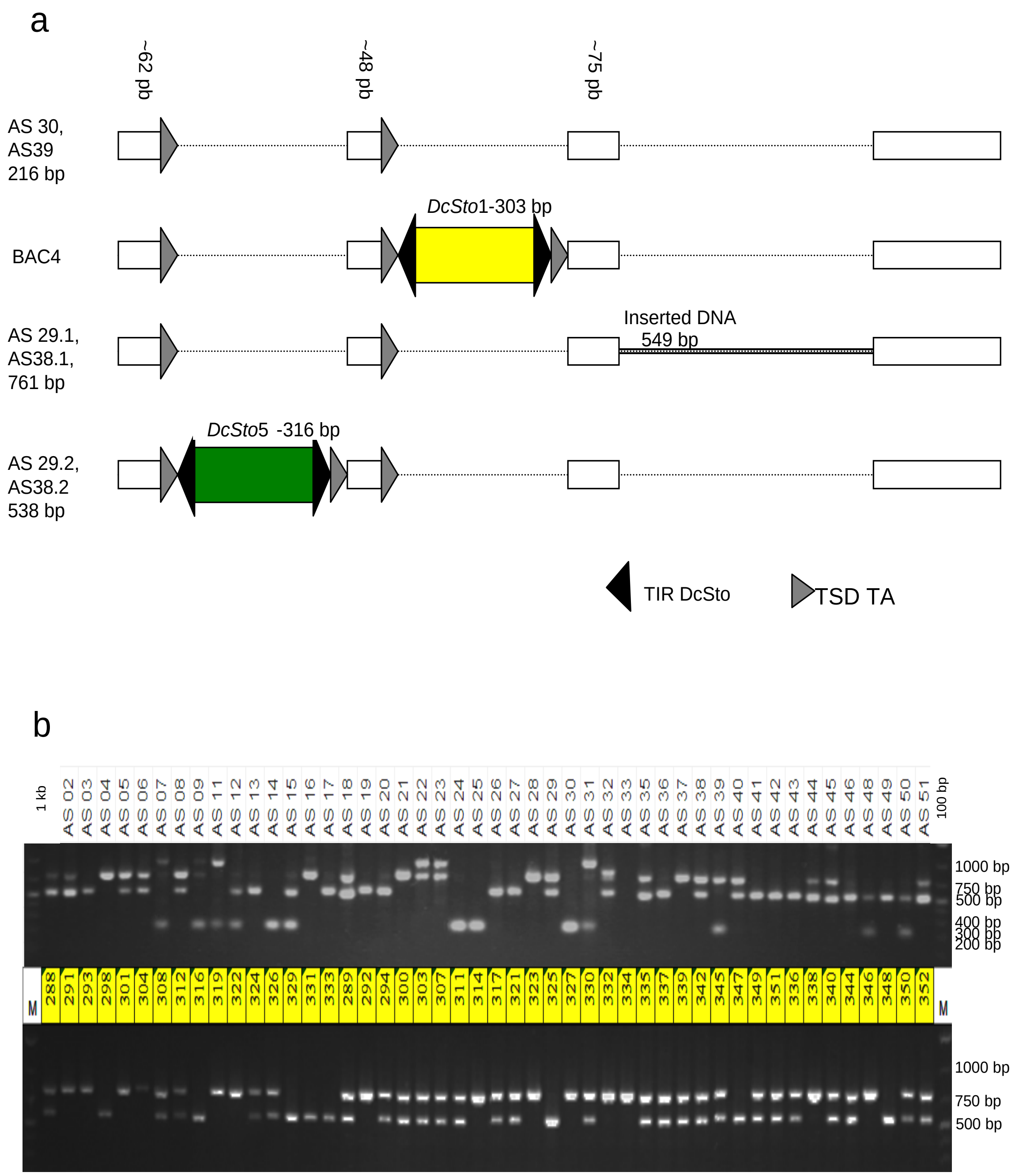

Supplemental Figure 4. A schematic representation of structural variants identified in BS4 locus (**a**), amplification profiles for a collection of plant representing the diversity of cultivated carrot (**b**). White box represents DNA flanking insertion site and dotted line represents gaps.

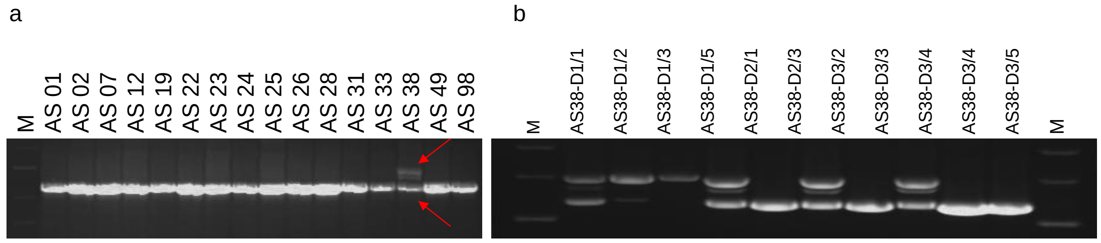

Supplemental Figure 5. Insertion of *DcSto*2 in the *chxb1* gene of carrot identified in one individual from the diversity collection (**a**) and segregating in the AS38 population (**b**) Allelic variants, corresponding to *chxb1* with and without the insertion are indicated with red arrows.

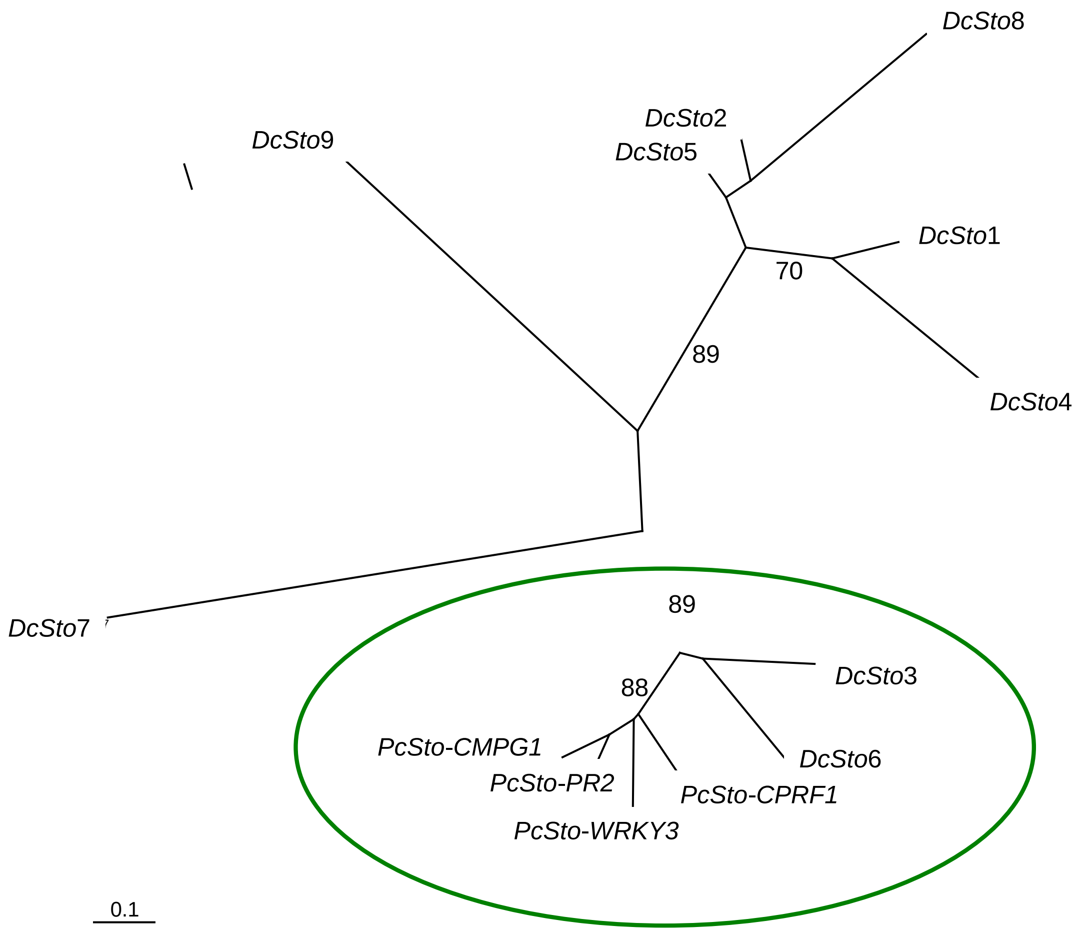

Supplemental Figure 6. Neighbor Joining tree showing relationships among parsley *PcSto* and carrot *DcSto* consensus sequences. The cluster grouping parsley and carrot elements is circled.
